# A Computational Modeling for Reconfigurable Biosensors

**DOI:** 10.1007/s12668-026-02504-w

**Published:** 2026-03-17

**Authors:** Roberta Grasso, Jose M. Gonzalez-Medina, Gian Luca Barbruni, Sandro Carrara

**Affiliations:** 1 Bio/CMOS Interfaces Laboratory, EPFL, Neuchatel, 2000 Switzerland; 2 Global TCAD Solutions GmbH (GTS), Wien, 1010 Austria

**Keywords:** Sensors, Molecular dynamic simulation, TCAD, Silicon nanowire, Aptamer, Reconfigurable transistor

## Abstract

For the first time ever reported, we present a multiscale modeling approach combining molecular dynamics simulations of probe-target binding with TCAD simulations of Reconfigurable Field Effect Transistors (RFETs). Field-effect transistor biosensors detect biomolecules by channel surface potential as affected by analyte charge. However, fixed channel doping limits them to sense either positive or negative targets. RFETs, based on doping-free nanowires, overcome this limitation by switching dynamically between n- and p-type modes. Recently proposed as a novel class of reconfigurable devices, RFETs remain almost unexplored as biosensors. Their intrinsic reconfigurability and high surface-to-volume ratio make them ideal for dual-polarity and high-sensitivity detection. To demonstrate RFET adaptability, we use both negatively and positively charged analytes, with two distinct recognition elements - aptamer and enzyme - highlighting the device’s ability to detect targets of opposite polarity through different binding mechanisms. The proposed multiscale framework establishes a direct link between molecular-scale binding phenomena and device-level electrical response, providing mechanistic insight into the sensing process and supporting the rational design of RFET-based biosensors. More broadly, the proposed methodology applies to a wide range of charge-based field-effect biosensors and supports device optimization and the prediction of experimentally observable trends.

## Introduction

FET biosensors incorporating nanomaterials as channel materials have demonstrated high sensitivity and selectivity across a broad range of targets, including SARS-CoV-2 [1], antibiotics [2], cancer biomarkers [3], and heavy metal ions [4]. The typical detection mechanism in FET-based biosensors relies on the modulation of the transducer’s conductance upon binding of a target biomolecule to the gate or to a receptor immobilized on it. This binding event alters the local electric field at the gate, which in turn modulates the drain current of the FET. By monitoring this change in current, the interaction of the target molecule with the receptor is detected. However, a fundamental limitation of conventional FET-based biosensors lies in their fixed polarity: the devices doped as either n-type or p-type depending on the expected charge of the analyte or the pH range conditions. This constraint reduces the flexibility of the sensing platform and limits its applicability in detecting a broader range of targets and in different media. A more scalable and versatile solution looks to detect both positively and negatively charged analytes within a single device architecture.

To this aim, Reconfigurable Field-Effect Transistors (RFETs) are considered. A RFET is an electronic device capable of reversibly switching between n-type and p-type operation. Unlike conventional field-effect transistors, which rely on impurity-based chemical doping, RFETs operate through electrostatic doping, whereby mobile charge carriers are induced by an externally applied voltage [5]. Typically, RFETs incorporate at least two independently controlled gates. The program gate (PG) selects the type of charge carriers (electrons or holes), while the control gate (CG) modulates the channel conductance and, consequently, the drain current. In contrast to conventional FETs, which are inherently unipolar as a result of their fabrication process, RFETs enable static or dynamic reconfiguration during operation. RFETs have recently emerged as promising candidates to overcome limitations of conventional FETs in several application domains, including reduced area and activity in adder architectures [6, 7], mitigation of charge-sharing effects in dynamic logic [8, 9], support for analog and mixed-signal circuit design [10], cryogenic CMOS hardware security [10], and neuromorphic computing [10]. Although their application in biosensing has not yet been experimentally demonstrated, RFETs represent promising candidates for this field. In this work, RFETs are investigated as a sensing platform capable of detecting both positively and negatively charged analytes using the same device, by dynamically switching between n-type and p-type operation through bias polarity reversal [11]. In addition, their nanowire-based architecture provides a high surface-to-volume ratio, which enhances sensitivity to variations in surface potential induced by molecular binding events and supports detection over a broad concentration range.

In the context of this study, dual-gated RFET devices are considered. In these devices, the specific placement of the program gate defines two commonly adopted configurations, namely the program-gate-at-drain (PGAD) and the program-gate-at-source (PGAS) configurations [12]. The PGAS configuration exhibits steeper switching characteristics, comparable to those of conventional MOSFETs, but is affected by increased OFF-state leakage. Moreover, the two configurations exhibit markedly different nonlinear behavior in the output characteristics, with a more pronounced nonlinearity observed in the PGAS case [13]. To limit leakage-induced sensitivity degradation and to reduce output nonlinearity, the PGAD configuration is adopted in this work.

In biosensing equally critical is the choice of recognition element, which must exhibit high affinity, specificity, and stability toward the target analyte. Different types of recognition elements rely on distinct molecular interactions, yet are often described within the framework of the lock-and-key model [14, 15]. For instance, in enzymes–ligand interactions, the enzyme selectively recognizes the shape of its substrate and transiently holds it in place at a specific region known as the active site. The active site is the part of the enzyme that binds the substrate to carry out the enzymatic reaction [14]. Aptamers, likewise, rely on structural flexibility and undergo conformational changes upon target binding [15]. These folding dynamics are crucial for the high specificity and affinity that characterize aptamer-based recognition.

Despite increasing use of Technology Computer Aided Design (TCAD) tools to model emerging biosensors, especially those involving nanometric devices with complex physics, analyte–probe interactions are typically modeled as static surface charges or effective potential shifts [16, 17]. This simplification fails to capture the dynamic and spatial complexity of the molecular interface, which can significantly affect charge transport and overall device behavior.

For example, although the small size of probes helps mitigate Debye screening effects, it does not eliminate them. Binding of a target molecule induces conformational rearrangements in the probe structure, potentially shifting the effective binding site outside the conventional Debye length thereby altering the local electrostatic environment. As a result, the sensor’s electrical response reflects not just the target concentration, but a combination of spatial rearrangement, charge redistribution, and analyte concentration. Because probe–target interactions are rarely modeled with sufficient rigor and are often interpreted empirically [18, 19], it becomes difficult to predict or optimize the sensor behavior. This lack of mechanistic understanding limits the ability to design biosensors with correct and tunable performance.

X-ray crystallographic analysis of probe–target complexes has traditionally been used to elucidate structural conformations and generate predictive models of binding-induced changes [20]. To gain insight into conformational dynamics even in the absence of crystallographic data, molecular modeling approaches have been developed. Among these, Molecular Dynamics (MD) simulations offer a powerful tool by capturing atomistic interactions, conformational flexibility, and solvent effects over time [21–24]. *Stuber et al.* [25] already proved that MD simulations can complement experimental work by revealing that target-induced structural rearrangements, not analyte charge alone, are the primary drivers of signal transduction. Their work further correlates aptamer height changes to electrochemical sensor responses, underscoring the impact of aptamer dynamics on signal generation. In addition to elucidating binding mechanisms, MD simulations enable predictive optimization of biosensor design, such as identifying optimal molecular density and orientation on the sensor surface [26]. While numerous experimental studies have examined how molecular surface density affects sensor sensitivity and detection limits [19, 27, 28], they typically rely on laborious procedures and yield limited mechanistic insights. In contrast, MD approaches have proven capable of identifying optimal densities more rigorously and unveiling experimental findings theoretically. [26, 29–32]

Therefore, integrating such molecular-level insights into TCAD simulations enables a powerful multiscale modeling framework that captures the full complexity of biosensing mechanisms.

To demonstrate this strategy, we explore two types of probes as recognition molecules: aptamers and enzymes. This dual approach is proposed here in order to demonstrate the versatility of our RFET-based biosensor in detecting both negatively and positively charged analytes using a single device. For the enzymatic recognition, structural information is derived from existing X-ray crystallographic data of enzyme–inhibitor complexes. For aptamers, due to the limited availability of structural databases, MD simulations are employed to capture dynamic conformational and electrostatic changes upon target binding.

As model of target molecule for the aptamer-functionalized RFET biosensor, carcinoembryonic enzyme (CEA) was selected due to its well-established role as a biomarker for the early detection of colorectal cancer - currently the third most common cancer in men and the second in women worldwide [33, 34]. For the enzyme-functionalized RFET biosensor, HIV-1 protease was chosen as the model probe, given its essential role in processing viral polyprotein precursors and its status as a key target in anti-AIDS drug development [35].

The innovation of this work lies in several key contributions. First, we present a generalizable multiscale modeling methodology for the design and optimization of biosensors, applicable across diverse sensor architectures. Second, we demonstrate the feasibility of using RFETs in biosensing, showing that their sensing behavior can be dynamically tuned via local electrostatic gating of Schottky barriers to detect both positively and negatively charged analytes. By incorporating molecular-level insights into TCAD simulations, we lay the theoretical groundwork for the deployment of RFETs in biosensing, enabling the development of next-generation, compact, and reconfigurable biosensor systems.

## Materials and Methods

### Molecular Simulations

The 2D structure of CEA was obtained from the PubChem website (https://pubchem.ncbi.nlm.nih.gov/) (CID: 10306739) and then converted in a 3D Structure via OpenBabel website [36].

The unbound structure of HIV-1 protease was obtained from the RCSB Protein Data Bank [37] (PDB ID: 1HHP), while the inhibitor-bound configuration, complexed with a nonpeptidic cyclic urea inhibitor ([4R-(4*α *,5*α *,6*β *,7*β *)]-hexahydro-5,6-dihydroxy-1,3-bis[2-naphthylmethyl]-4,7-bis(phenylmethyl)-2H-1,3-diazepin-2-one), was obtained from the PDB entry 1HVR, based on the work of *Lam et al.* [38].

A DNA anti-CEA aptamer with sequence 5’-GGGAACCATCGA- GTTACACCGACCTT- CTATGTGCGGCCCC-3’ was selected based on its reported high affinity for CEA [39]. The 2D and 3D structures of the selected aptamer were predicted using the mfold web server [40] and 3dRNA/DNA web server [41] respectively.

A thiol group was introduced at the 5’ end to simulate surface immobilization using the Molefacture plugin in the Visual Molecular Dynamics (VMD) software [42]. The modified aptamer was subsequently tethered to a gold slab to reproduce the experimental surface-immobilization conditions, using the Inorganic Builder plugin in VMD [43]. To investigate the influence of surface probe density, multiple simulations were conducted with varying numbers of aptamer molecules. The simulation box size was kept constant and sufficiently large to accommodate the highest tested surface density, while only the number of inserted aptamers was modified using the insert-molecules [44] tool in GROMACS. Although this approach increased the computational burden due to a larger atom count and box size, it was essential for ensuring a fair comparison across densities, particularly in terms of steric hindrance and system stability.

The above protocol describes the case of the aptamer in its unbound (free) state; for simulations involving the bound configuration, molecular docking was performed using AutoDock Vina (v1.2.1) [45] and the resulting complex was used as the starting configuration for subsequent molecular dynamics simulations (MDS). Energy minimization was performed using GROMACS 2021.4 [46] with the AMBER99SB-ILDN force field. Equilibration was carried out in both NVT (constant number of particles, volume, and temperature) and NPT (constant number of particles, pressure, and temperature) ensembles for 100 ps each, using a 2 fs time step. The production simulation was run for 2000ns at room temperature (298 K) and 1.0 atm. Post-simulation, GROMACS analysis tools were used to evaluate the system’s conformational dynamics. Root-mean-square deviation (RMSD) and radius of gyration ($R_g$) analyses were performed to assess structural stability. These metrics were also used to determine the optimal surface density of aptamers, defined as the configuration that minimized RMSD while maintaining overall system integrity.

The binding free energies were calculated using the Molecular Mechanics Poisson-Boltzmann Surface Area (MM-PBSA) method [47]. The MMPBSA analysis provided detailed contributions to the binding free energy from van der Waals interactions, electrostatic interactions, and polar solvation energies.

### TCAD Simulation

**Fig. 1:**
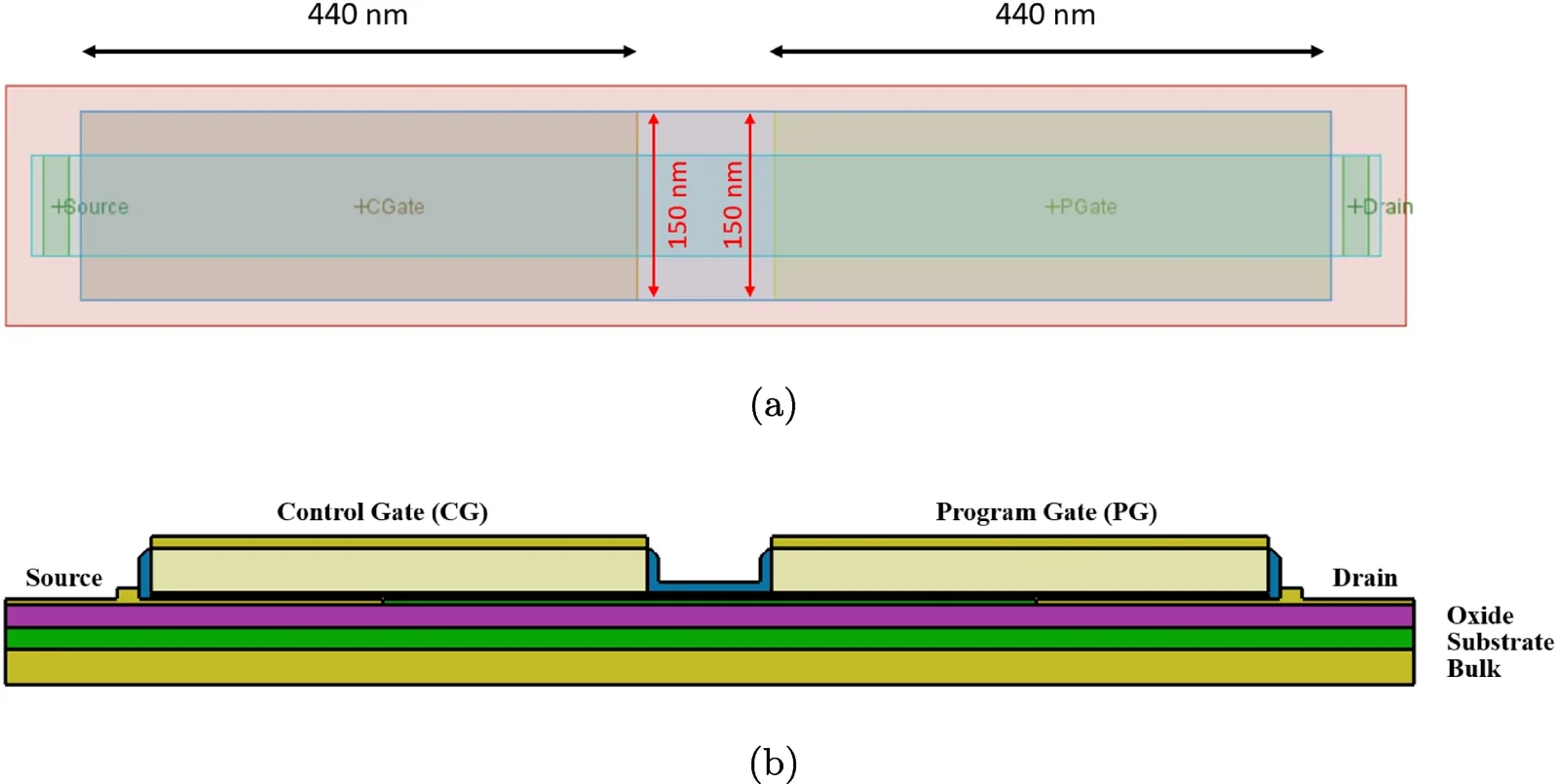
**a**) Top view and **b**) Front view of the simulated RFET device

TCAD simulations were conducted using the commercial simulator Minimos-NT [48], part of Global TCAD Solutions (GTS) Framework. The simulated device is a dual-gate RFET, featuring a program gate (PG) located above the drain contact and a control gate (CG) positioned over the source contact (PGAD configuration) as shown in Fig. 1a. The device features a 1 µm-long doping-free ultra-thin silicon channel [5, 49]. Both the control gate and the program gate are 440 nm in length. A High-K Gate Metal (HKMG) stack is used to ensure strong gate electrostatic control. The gate width is 150 nm, yielding to an active sensing area of 66,000 nm$^{2}$. A front-view cross-section of the device is shown in Fig. 1b.

The simulations employed the semi-classical drift-diffusion (DD) transport model to describe carrier dynamics within the channel, while accounting for the effects of quantum confinement by means of the density gradient model [50]. The charge injection and extraction at the Schottky junctions were modeled using the Tsu Esaki formalism to account for thermionic emission and quantum mechanical tunneling [51].

In the simulation protocol, the PG is biased with a charge input to emulate molecular functionalization, while the CG voltage is swept to extract the device’s transfer characteristics.

## Results and Discussion

### Aptamer-CEA Molecular Results

MM-PBSA simulations on the CEA-specific aptamer and its target estimated a mean binding free energy of -12.05 kcal/mol, corresponding to a dissociation constant ($K_D$) of 1.47 nM. This value aligns with previously reported $K_D$ values for other CEA-specific aptamers [52] and is consistent with the affinity observed for the same aptamer [39] in studies involving T84 cells, which express the CEA protein.

The binding affinity of aptamers is typically reported in the nanomolar to micromolar range [53–55]. Specifically, previous studies investigating anti-CEA aptamer interactions have focused on target concentrations within the nanomolar range [39, 52]. To ensure consistency with these established benchmarks and to evaluate sensor performance under challenging, low-concentration conditions, the simulations in this work were conducted across a range from 0 to 100 nM.

Experimental evidence highlights the importance of aptamer surface density in determining biosensor performance, as it directly impacts sensitivity and detection limits. [28, 56]. Reported aptamer probe densities commonly range from $10^{11}$ to $10^{13}$ molecules/cm$^{2}$ for biological medium [57–59], depending on factors such as target size, steric hindrance, and electrostatic repulsion. Given the predicted aptamer–target size and the available gate sensing area, the maximum achievable density in our system was estimated to be $1.5 \times 10^{12}$ molecules/cm$^{2}$.

**Fig. 2:**
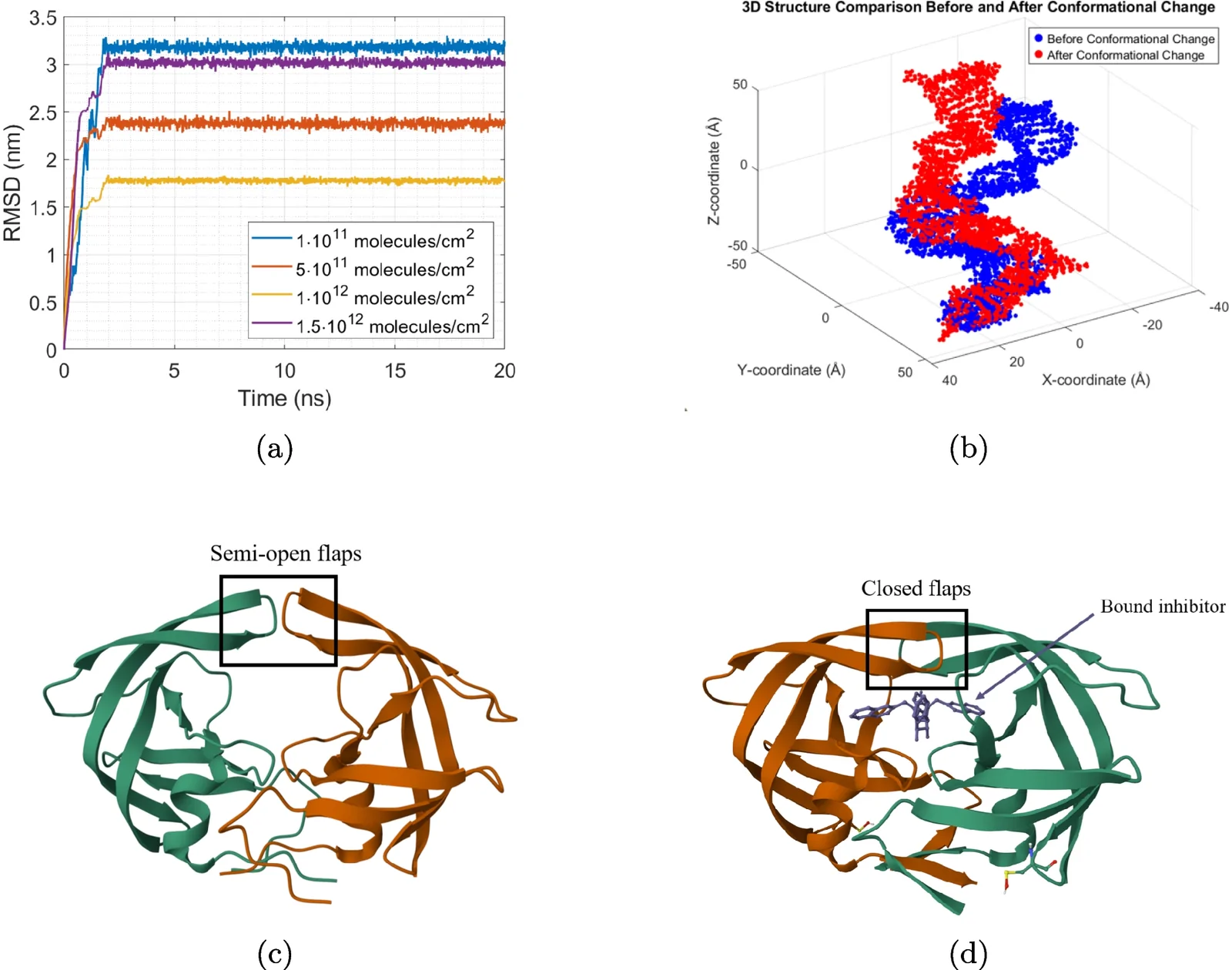
**a**) RMSD values for the aptamer-target complex over the first 20 ns of simulation. Early convergence is evident from 2 ns, with minimal fluctuations observed throughout. A stable RMSD of approximately 1.7 nm at a surface density of $10^{12}$ molecules/cm$^{2}$ indicates the optimal aptamer packing on the substrate. **b**) Comparison of the aptamer’s 3D structure before (blue) and after (red) binding to the target molecule. Upon binding, the aptamer undergoes a conformational rearrangement, elongating along the z-axis as a result of the “lock-and-key” interaction mechanism. **c**) The unbound HIV-1 protease is shown in the semi-open flap conformation (PDB ID: 1HHP) while **d**) the inhibitor-bound form adopts a fully closed flap configuration (PDB ID: 1HVR). Notably, the handedness of the flaps differs between the two states and is indicated above each structure. The flap tips (residues 49–52) are highlighted within the black box

To identify the optimal surface density, MD simulations were conducted at four aptamer densities: $10^{11}$, $5 \times 10^{11}$, $10^{12}$, and $1.5 \times 10^{12}$ molecules/cm$^{2}$. The RMSD was computed over a 2 *μ *s simulation period to evaluate the conformational stability of immobilized aptamers. Steric hindrance was assessed by monitoring the accessibility of the predicted binding pocket across all configurations. Since the pocket remained consistently exposed and unobstructed, the final density was selected based on minimizing RMSD. As shown in Fig. 2a, RMSD trajectories reached equilibrium with fluctuations within 2 Å after approximately 2 ns, indicating system convergence. The configuration at $10^{12}$ molecules/cm$^{2}$ exhibited the lowest RMSD, suggesting an optimal balance between structural stability and minimized electrostatic repulsion.

Trajectory analysis further reveales a notable conformational change upon target binding: the aptamer elongates in the z-direction, effectively displacing its negatively charged phosphate backbone away from the substrate. Specifically, the aptamer’s height, measured as z-distance from the gold slab, increased from 7.15 nm (unbound) to 9.13 nm (bound) (Fig. 2b). Binding events occurred approximately 3.4 nm above the substrate surface, which lies beyond the Debye length in 1*× *PBS but within range in 0.01*× *PBS. This finding shows the importance of operating in low-ionic-strength or dry conditions to enable effective detection, and demonstrates the value of molecular dynamics simulations in guiding experimental setup by providing predictive insights from theoretical analyses.

### HIV-1 Protease-Inhibitor Molecular Results

In the HIV-1 protease case study, the $K_D$ between the protease and its inhibitor has been experimentally reported as 0.31 nM [60]. The conformational rearrangement following inhibitor binding is well established in the literature. Indeed, extensive X-ray crystallographic studies have revealed that HIV-1 protease forms a C2-symmetric homodimer with a prominent substrate-binding pocket shielded by two glycine-rich *β *-hairpin flaps [61–64]. A consistent structural shift is observed between the bound and unbound states: in the liganded form, the flaps adopt a fully “closed” conformation, drawn tightly over the active site, while in the unbound state they assume a “semiopen” conformation, positioned further from the catalytic Asp-25–Thr-26–Gly-27 triads yet still partially covering the binding pocket [60] (Fig. 2c and d). Notably, the “handedness” of the flaps is reversed between the two states, whereas the rest of the structure remains largely unchanged [60].

To maintain consistency with the CEA simulations, the enzyme surface density was set to $10^{12}$ molecules/cm$^{2}$, and the inhibitor concentration was varied from 0 to 100 nM.

### Binding Curve Extraction

To model probe–target interactions in both case studies, we employed the single-site binding approximation [65, 66]. Although this model assumes a simplified 1:1 binding stoichiometry at equilibrium, thereby neglecting potential multi-valent or cooperative effects, it remains one of the most robust and experimentally supported frameworks for describing both aptamer and enzyme–mediated recognition [67–70]. Its validity across diverse biosensing platforms and its strong agreement with experimental data justify its widespread adoption.

**Fig. 3:**
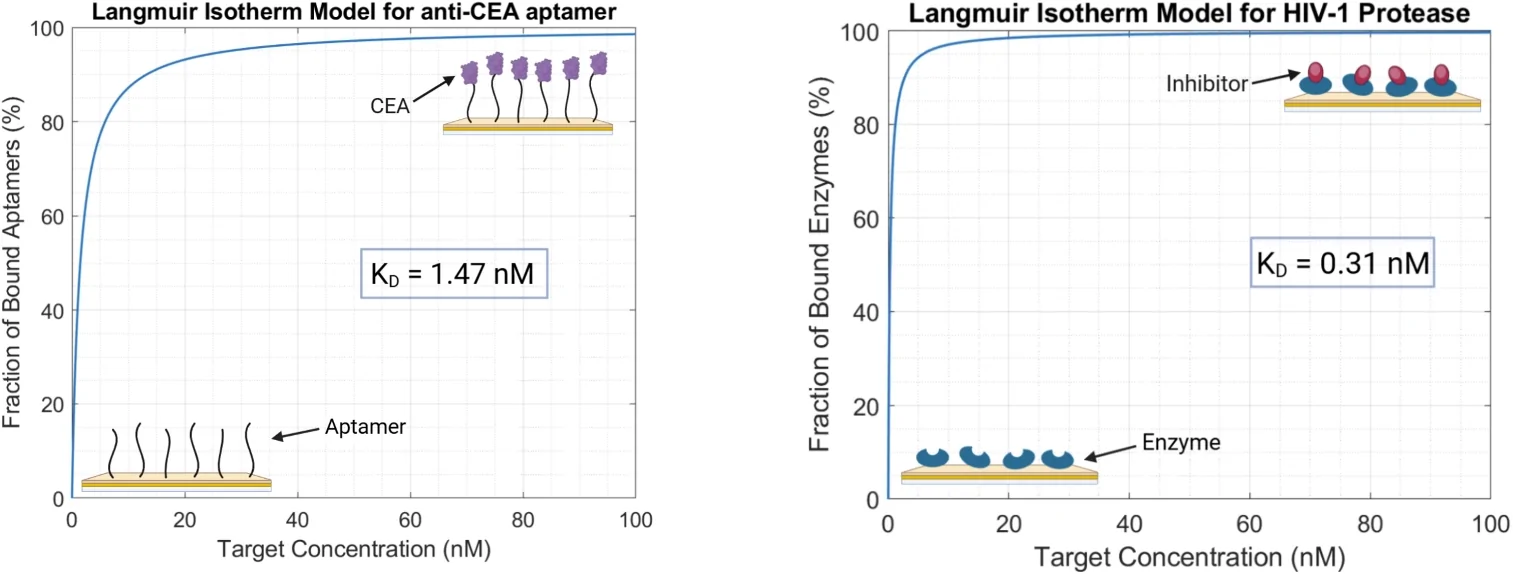
Langmuir isotherms describing the binding equilibrium between: (**a**) aptamer and CEA, and (**b**) enzyme and inhibitor. The model assumes a 1:1 binding stoichiometry at equilibrium. The fraction of bound probes depends on both the target concentration and the dissociation constant, $K_D$. As the target concentration increases, a growing number of probes become occupied until saturation is reached, where all available binding sites are fully engaged by target molecules

This approximation is grounded in Michaelis–Menten kinetics and yields the classical Langmuir isotherm (Eq. 1):

1 $$\begin{aligned} f_a = \frac{[T]}{K_D + [T]} \end{aligned}$$

with $f_a$ being the fraction of bound probes, [T] the free target concentration, and $K_D$ the dissociation constant. The equation outputs the form of a rectangular hyperbola in which ends with a curve infinitely nearing a constant value (asymptote of 1) at the point of saturation of the binding sites. From this binding curve, $K_D$ values of the system can be estimated as the curve crosses the point of $f_a$ = 0.5 and is defined as the target concentration at which 50% of the probe molecule is bound to the target under equilibrium conditions. The resulting binding curves for both the aptamer–target and enzyme–target interactions are presented in Fig. 3.

### TCAD Results

**Fig. 4:**
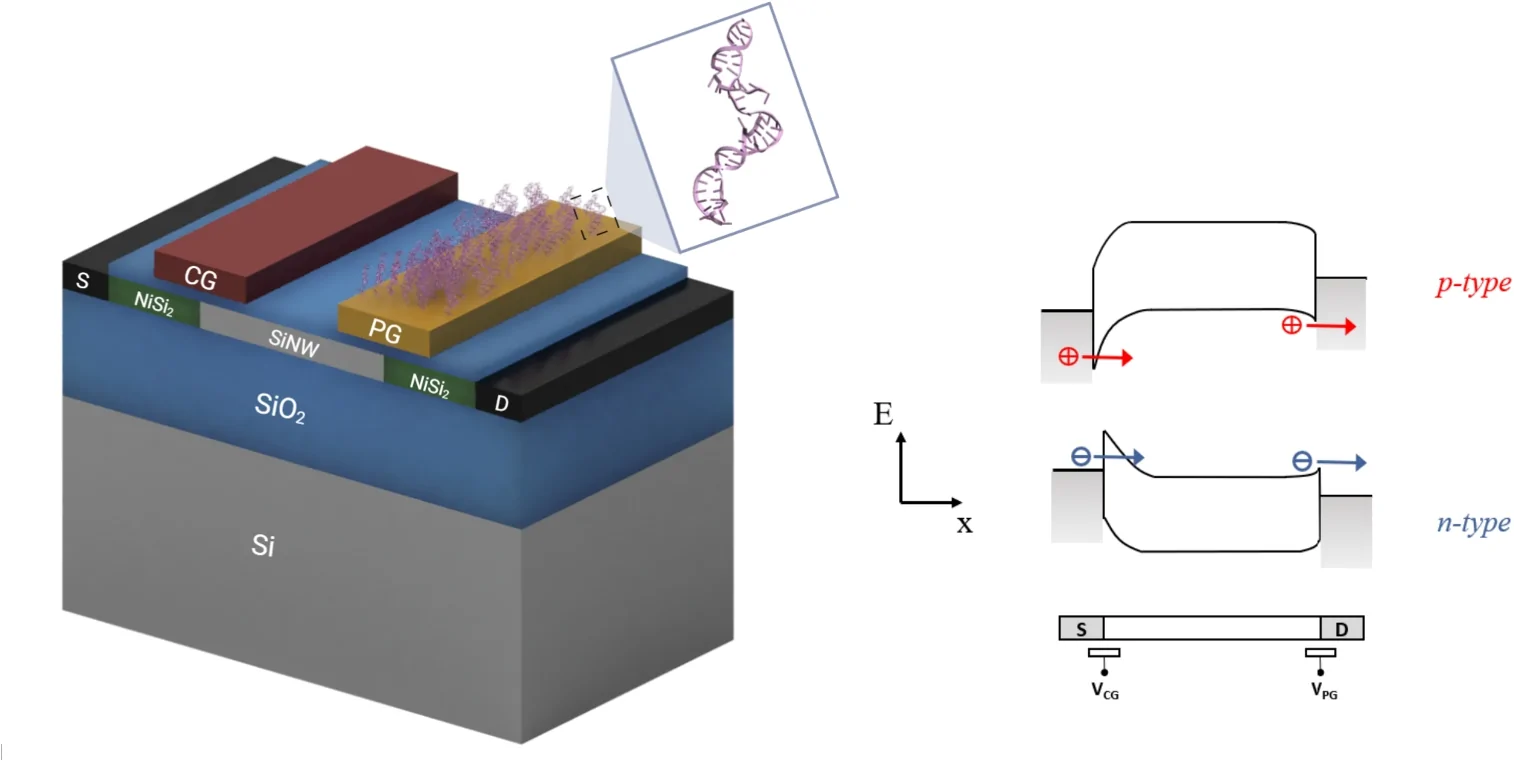
**a**) Schematic of the simulated Dual-Gate RFET structure. The PG at the drain Schottky junction determines the carrier polarity, the voltage of the CG facing the source switches the transistor ON and OFF. Probes are positioned in the program gate and the change in the drain current is evaluated for different target concentrations; **b**) Schematic band diagrams of the RFET in the ON state for p-type (up) and n-type (down) configuration

Figure 4 shows the simulated PGAD device structure along with the corresponding energy band diagrams. As previously mentioned, the PG, positioned at the drain-side Schottky junction, defines the device’s conduction type by electrostatically suppressing the unwanted carrier species. The CG is located at the source-side junction and modulates the injection efficiency of the desired carrier type into the channel. [10].

To achieve electron-dominated (n-type) conduction, a positive voltage applied to the program gate ($V_\textrm{PG} > 0$ V) lowers the valence band edge ($E_V$) at the drain-side junction, effectively blocking hole injection. Simultaneously, the control gate voltage ($V_\textrm{CG}$) modifies the screening length of the Shottky barrier heigth at the source, facilitating electron injection into the channel. In contrast, hole-dominated (p-type) conduction is programmed by applying negative voltages to both gates ($V_\textrm{PG} < 0$ V and $V_\textrm{CG} < 0$ V). This configuration causes upward band bending at both junctions: the drain-side conduction band is raised to suppress electrons, while the source-side Schottky barrier becomes thinner, thereby promoting hole injection from the source. [11]

To assess the impact of molecular functionalization on device performance, TCAD simulations incorporated the electrostatic profiles of the probes in both their unbound and target-bound states onto the PG. These charge distributions were derived from the molecular conformations in both bound and unbound states - extracted from X-ray crystallographic data in the case of the enzyme–inhibitor complex, and from MD simulations for the aptamer–target system.

Using the simulated optimal probe density ($10^{12}$ molecules/cm$^{2}$), the corresponding number of molecules was calculated and applied as a reference in the device-level modeling. Since the electrostatic signature differs between the unbound and target-bound configurations, simulations were carried out across a range of binding scenarios. Specifically, the fraction of bound probes was systematically varied from 0 % (all probes unbound) to 100 % (all probes bound), in order to emulate realistic sensing conditions in which only a subset of surface-immobilized probes is engaged with the target, depending on its concentration. For each simulated condition, the total surface charge was modeled as a weighted combination of the bound and unbound charge distributions. These mixed profiles were applied to the program gate of the RFET to demonstrate the driving effect on the device’s electrical characteristics.

At physiological pH, CEA carries a slight negative charge due to the presence of sialic acid residues in its glycan chains [71]. Additionally, the aptamer itself is negatively charged, owing to its phosphate backbone [72]. When the charge distributions of both the free aptamer and the aptamer–target complex are introduced at the PG of the RFET, a virtual p-type doping effect is induced in the drain region. Simultaneously, the carrier injection at the source-side Schottky barrier is modulated by varying values of the control gate voltage ($V_{CG}$). The transfer characteristics (I_D_-V_GS_ curves) were simulated by stepping the control gate voltage in the negative range from -5 to 0 V at a fixed drain-source bias of -0.1 V.

**Fig. 5:**
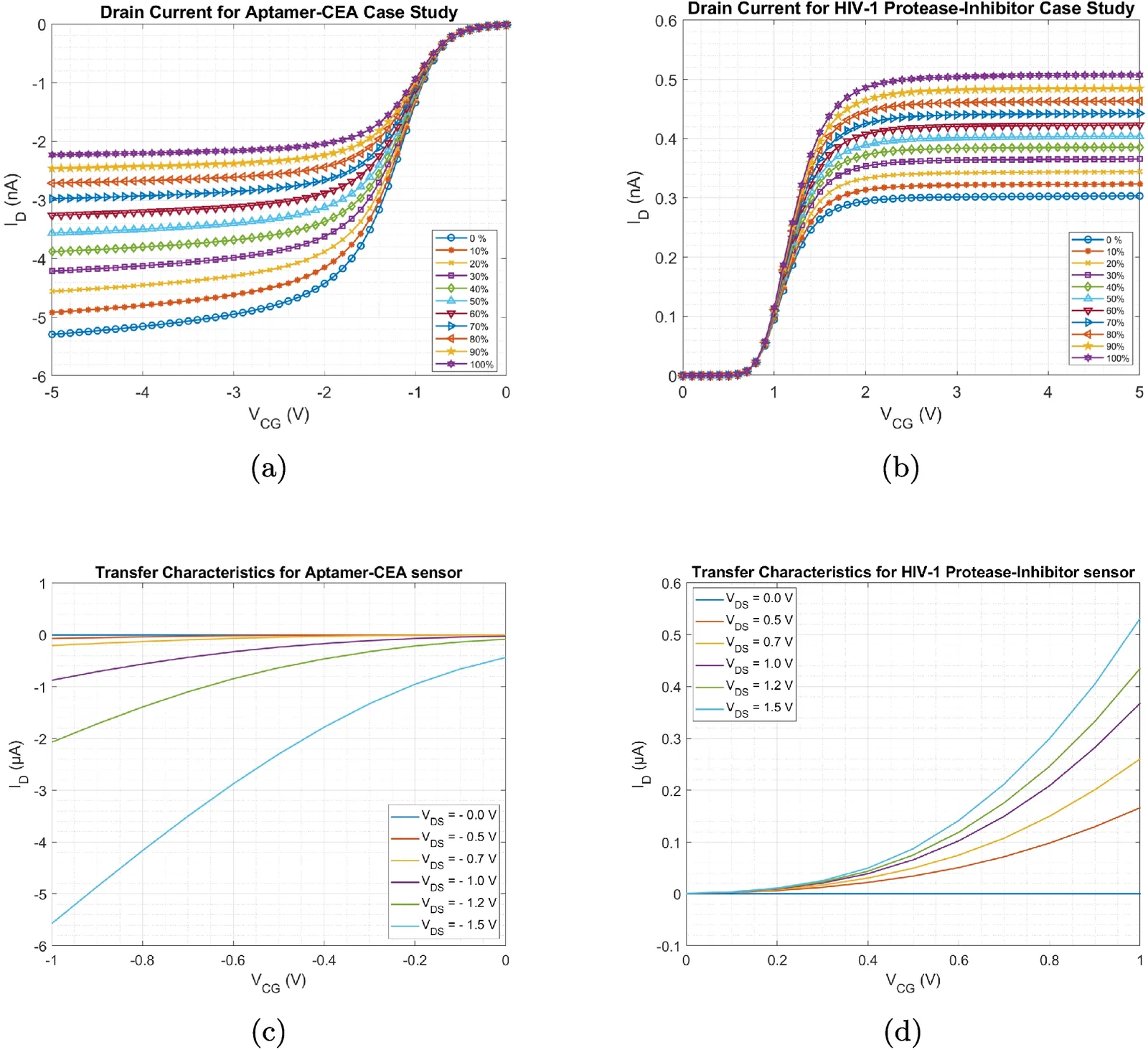
Drain current as a function of biomolecular interactions. (**a**) As the fraction of bound aptamers increases, the drain negative current increases due to aptamer elongation reducing electrostatic influence on the program gate. (**b**) An increase in $I_{D}$ is observed with bound enzymes owing to the more positive net charge with respect to free enzyme. (**c**) Transfer characteristics ($I_{D}$–$V_{GS}$) of the RFET-based aptasensor for increasing $|V_{DS}|$, with a fixed bound aptamer fraction of 10%. (**d**) Transfer characteristics of the RFET-based enzyme sensor for increasing $V_{DS}$ at a fixed 10% bound enzyme fraction

Although CEA is a negatively charged target and an increase in current might therefore be expected due to enhanced electrostatic gating, the binding event instead induces a conformational elongation of the aptamer, as previously demonstrated by molecular dynamics simulations (MDS). This structural rearrangement shifts the negatively charged phosphate backbone farther from the channel, reducing its electrostatic coupling with the substrate. In p-type operation, this diminished electrostatic influence results in reduced hole accumulation, due to an upward shift of the energy bands at the drain-side Schottky barrier. Consequently, as the fraction of bound aptamers increases, the drain current becomes smaller, as shown in Fig. 5a. Moreover, increasing $V_{CG}$ raises the valence band at the source-side Schottky contact, enhancing tunneling and thus further contributing to the increase in current.

In the case of HIV-1 protease, the overall surface charge distribution is shaped by the presence of charged amino acids and ligand binding. Notably, a positively charged cluster centered around residue 69 has been identified [73]. Upon inhibitor binding, the total charge of the resulting complex increases, remaining positive but with a greater magnitude than that of the free protease, due to the inherent positive charge of the inhibitor. Accordingly, when this charge distribution is incorporated into the RFET model, an n-type FET behavior emerges. For this reason, the transfer characteristics (I_D_-V_GS_ curves) were simulated, in this case, by stepping the control gate voltage in the positive range from 0 V to 5 V at a fixed drain-source bias of 0.1 V. The presence of positively charged molecules near the program gate induces a downward shift in the conduction band at the source-side Schottky barrier, facilitating electron injection into the channel. As the proportion of bound protease–inhibitor complexes increases, this effect becomes more pronounced, leading to a corresponding rise in the drain current (Fig. 5b).

The lower current observed in this second case study is attributed to the comparatively weaker net charge of the enzyme–inhibitor complex, especially when contrasted with the strong negative charge of the aptamer in the aptamer–CEA system.

An increase in drain current is observed with higher $|V_{DS}|$ values in both configurations (Fig. 5c and d), attributed to reduced exit resistance at the drain-side Schottky barrier.

**Fig. 6:**
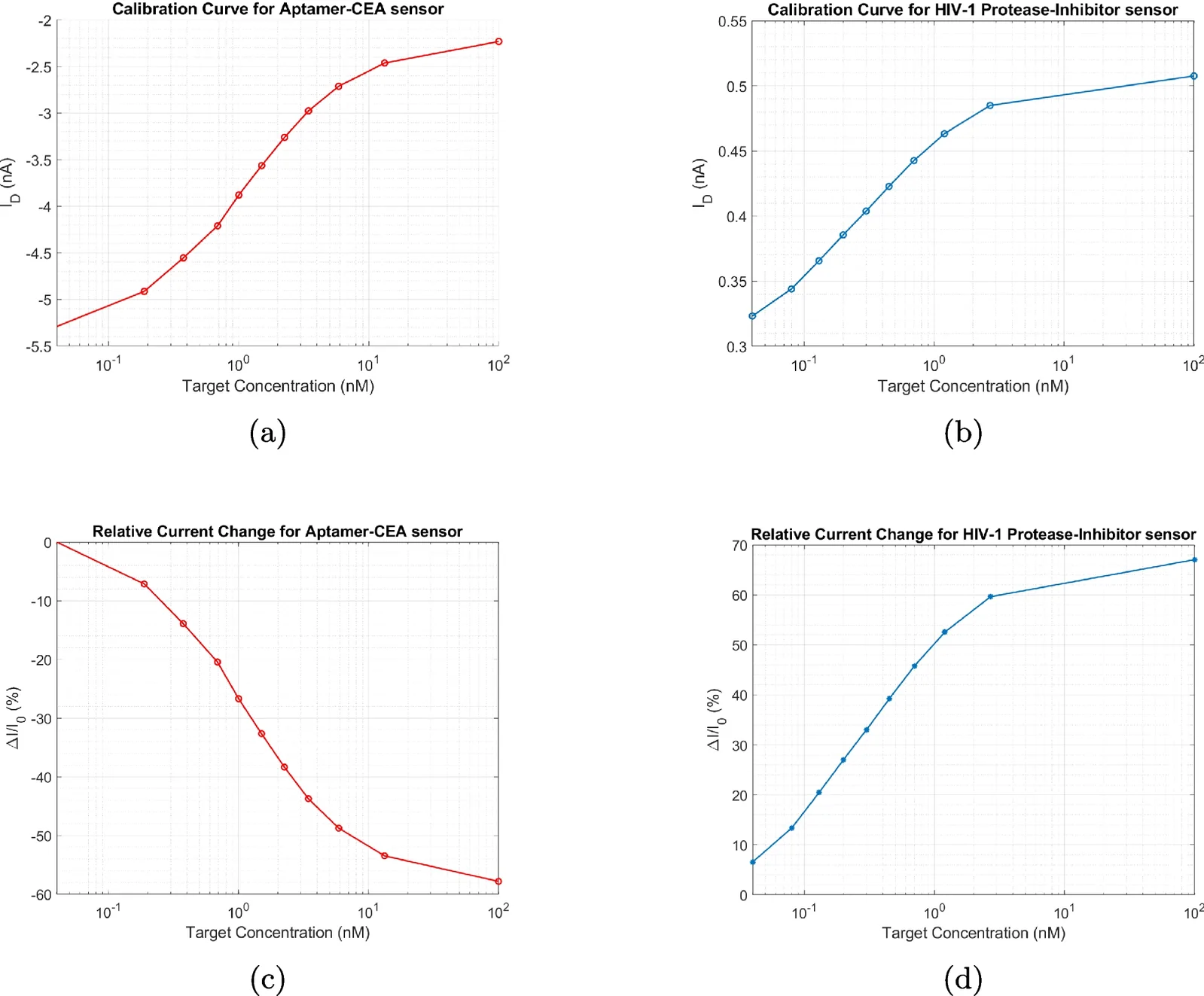
(**a**) Calibration curve of the aptasensor across the CEA concentration range. (**b**) Calibration curve of the enzyme sensor across the inhibitor concentration range. (**c**) Normalized current change for the Aptamer-CEA sensor. (**d**) Normalized current change for the HIV-1 Protease-Inhibitor sensor. All the curves are obtained with a fixed bias of $|V_{DS}|= 0.1 V$

By correlating the simulated drain current at varying fractions of bound aptamers and enzymes with the Langmuir adsorption curves (Fig. 3a and b), a direct relationship between device output (i.e., drain current) and target concentration is established. When $|V_{CG}|$ is set to 5 V and $|V_{DS}| = 0.1$ V, a non-linear calibration curve emerges for both the two cases (Fig. 6a and b).

The influence of target concentration differs between the two systems: in the aptamer–CEA case, binding induces elongation of the aptamer, which shifts its negatively charged backbone further from the surface, reducing the electrostatic modulation and thus decreasing the current with increasing target concentration. In contrast, for the HIV-1 protease case, binding increases the net positive charge near the program gate, enhancing electrostatic gating and thereby increasing the output current as target concentration rises.

To evaluate and better compare sensors’ detection sensitivity in these two cases, the relative current variation ($\Delta I / I_0$) was calculated, where $\Delta I = I_{D} - I_{0}$ represents the change in drain–source current. Here, $I_{D}$ refers to the current measured in the presence of the target analyte, and $I_{0}$ corresponds to the current in its absence. The RFET-based aptasensor for CEA demonstrated a maximum current suppression of 57% with increasing analyte concentration (Fig. 6c), while the HIV-1 Protease-based sensor exhibited a signal enhancement of up to 67% as the inhibitor concentration increased (Fig. 6d).

As demonstrated, the sensor exhibits a distinct response across clinically relevant concentration ranges for both CEA and inhibitor detection, underscoring the adaptability of the RFET platform for sensing analytes with varying charge profiles.

## Conclusion

In this work, we introduced a multiscale approach that bridges the gap between molecular biochemistry and device physics by integrating atomistic molecular data and dynamic simulations with TCAD device modeling for the new class of RFET biosensors. This combined framework guides sensor design, enables predictive insight into experimental outcomes, and clarifies underlying mechanisms, particularly for emerging devices such as RFET biosensors. For the HIV-1 protease system, X-ray crystallographic data provided detailed structural information and insights into conformational changes upon ligand binding. In contrast, the limited structural data available for aptamer–target complexes was addressed through MD simulations, which enabled the reconstruction and analysis of aptamer dynamics at the molecular level. Through MD, we quantified the interactions between aptamers, the target analytes, its substrate as well as neighboring probes. These simulations reveales the optimal surface density required to reduce steric hindrance and electrostatic repulsion and captured the structural rearrangements occurring upon target binding. Furthermore, they provided spatial localization of the binding sites with respect to the Debye screening length - crucial for effective sensor response. The resulting molecular insights were integrated into TCAD simulations, where charge redistribution was modeled as a dynamic process rather than a static surface charge, enabling a more accurate evaluation of the RFET’s electrical response to biomolecular interactions.

Thanks to the unique reconfigurability enabled by the program gate, RFETs allow the detection of analytes with varying charge polarity, while the control gate enables precise modulation of channel conductance over a wide range, supporting broad dynamic detection.

This works provides compelling evidence of the feasibility of using RFETs as adaptable biosensors. While the absolute accuracy of the simulated values will be verified in future experimental studies, the present results mark a significant relative advancement - establishing a solid foundation for the integration of RFETs into next-generation biosensing technologies. Although this work specifically addresses RFET-based biosensors, the proposed multiscale framework provides a general and transferable methodology for charge-based field-effect sensing platforms, enabling a unified modeling approach to link molecular interactions with device-level response across a broad range of biosensor technologies.

## Data Availability

Data are available from the authors upon request.

## Declarations

### Research Involving Humans and Animals Statement

This study did not involve human participants or animals.
